# YOLOv8-MFD: An Enhanced Detection Model for Pine Wilt Diseased Trees Using UAV Imagery

**DOI:** 10.3390/s25113315

**Published:** 2025-05-24

**Authors:** Hua Shi, Yonghang Wang, Xiaozhou Feng, Yufen Xie, Zhenhui Zhu, Hui Guo, Guofeng Jin

**Affiliations:** 1 College of Sciences, Xi’an Technological University, Xi’an 710021, China; 2 Shaanxi Academy of Forestry, Xi’an 710016, China; 3 Xi’an New Aomei Information Technology, Co., Ltd., Xi’an 710100, China

**Keywords:** pine wilt disease, UAV remote sensing, YOLOv8, MobileViT, focal modulation, dynamic head, forest pest and disease monitoring

## Abstract

Pine Wilt Disease (PWD) is a highly infectious and lethal disease that severely threatens global pine forest ecosystems and forestry economies. Early and accurate detection of infected trees is crucial to prevent large-scale outbreaks and support timely forest management. However, existing remote sensing-based detection models often struggle with performance degradation in complex environments, as well as a trade-off between detection accuracy and real-time efficiency. To address these challenges, we propose an improved object detection model, YOLOv8-MFD, designed for accurate and efficient detection of PWD-infected trees from UAV imagery. The model incorporates a MobileViT-based backbone that fuses convolutional neural networks with Transformer-based global modeling to enhance feature representation under complex forest backgrounds. To further improve robustness and precision, we integrate a Focal Modulation mechanism to suppress environmental interference and adopt a Dynamic Head to strengthen multi-scale object perception and adaptive feature fusion. Experimental results on a UAV-based forest dataset demonstrate that YOLOv8-MFD achieves a precision of 92.5%, a recall of 84.7%, an F1-score of 88.4%, and a mAP@0.5 of 88.2%. Compared to baseline models such as YOLOv8 and YOLOv10, our method achieves higher accuracy while maintaining acceptable computational cost (11.8 GFLOPs) and a compact model size (10.2 MB). Its inference speed is moderate and still suitable for real-time deployment. Overall, the proposed method offers a reliable solution for early-stage PWD monitoring across large forested areas, enabling more timely disease intervention and resource protection. Furthermore, its generalizable architecture holds promise for broader applications in forest health monitoring and agricultural disease detection.

## 1. Introduction

Pine Wilt Disease, caused by the pinewood nematode *Bursaphelenchus xylophilus*, is a highly lethal forest disease characterized by its rapid transmission and extensive impact. It has emerged as a significant threat to the ecological security of pine forests worldwide. In China, the disease spreads at an average rate exceeding 20 km per year, often leading to large-scale forest dieback within a short period [1]. Research indicates that PWD has resulted in severe losses of forest resources and ecosystem services, with direct economic losses in forest biomass estimated at USD 170 million and control expenditures reaching USD 940 million. Moreover, PWD poses a serious threat to biodiversity, causing pronounced degradation in the population structure of pine species in affected regions. These figures highlight that PWD is not merely a silvicultural issue, but a major ecological security challenge that urgently calls for interdisciplinary management strategies and scientifically informed interventions.

**Figure 1:**
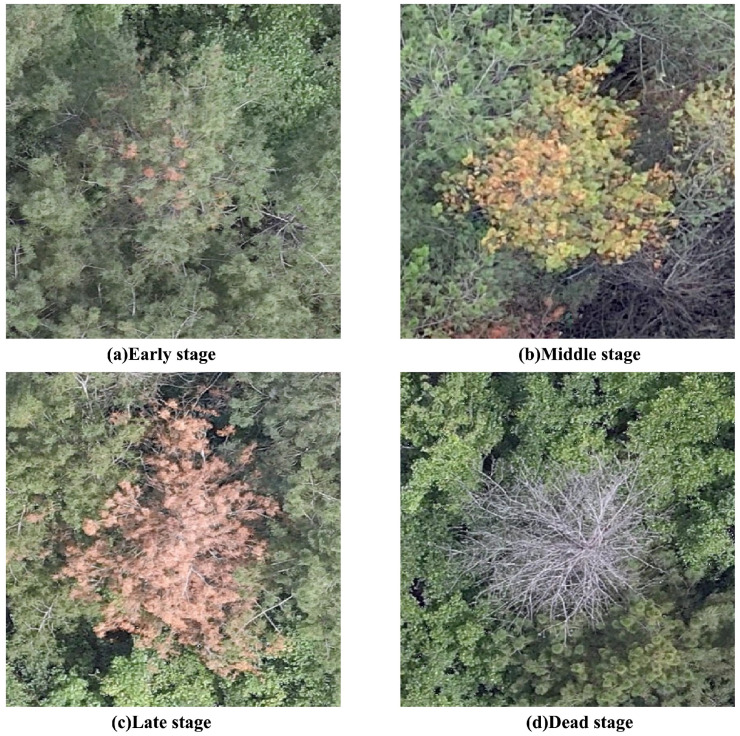
Diagram showing examples of different stages of PWD infection.

Currently, the identification of Pine Wilt Disease (PWD) primarily relies on monitoring color changes that occur during the progression of the disease. Figure 1 illustrates the distinct stages of PWD infection [2]: (a) In the early stage, following the invasion of Bursaphelenchus xylophilus into the pine trunk, the nematodes rapidly reproduce and spread throughout the host tree, damaging the xylem tissue and disrupting water transport and transpiration. (b) In the middle stage, as the water conduction system deteriorates, the tree begins to suffer dehydration, and some needles turn yellowish-brown. (c) In the late stage, all needles become yellow-brown, and cracks may appear on the trunk and bark; the discoloration then progresses to a reddish hue, with needles turning reddish-brown, expanding from localized areas to the entire canopy. (d) In the dead stage, the infected tree dies completely, with needle drop and the entire tree exhibiting a grayish-white, lifeless appearance. These characteristic color transitions provide critical visual cues for the accurate detection and diagnosis of PWD.

Currently, the detection of Pine Wilt Disease (PWD) primarily relies on deep learning methods. A considerable number of researchers employ the YOLO (You Only Look Once) series as the foundational framework, which is then optimized for the specific task of PWD detection in remote sensing imagery. To further enhance model performance, attention mechanisms are commonly introduced to improve the model’s ability to focus on critical regions, thereby increasing the accuracy of lesion feature extraction. Jianyi Su et al. [3] proposed a PWD-YOLOv8n-based detection model, which incorporates a Convolutional Block Attention Module (CBAM) and Coordinate Attention (CA) into the YOLOv8 backbone to strengthen feature representation. Additionally, the model integrates a Bidirectional Feature Pyramid Network (BiFPN) to improve the detection of small-scale diseased trees, thereby enhancing the model’s sensitivity to disease-related features. Zengjie Du et al. [4] introduced a model based on YOLOv5L-s-SimAM-ASFF, using YOLOv5L as the base architecture while replacing its original backbone with ShuffleNetV2. This model incorporates the SimAM attention module and the Adaptive Spatial Feature Fusion (ASFF) module to boost performance. It achieves high detection accuracy while maintaining a lightweight design, making it suitable for deployment on edge devices. In 2021, Xinquan Ye et al. [5] proposed an improved YOLOv5 [6] model that integrates the SOCA mechanism, which enhances the model’s ability to detect PWD-infected trees in complex forest environments and improves multi-scale feature extraction. This model is well suited for large-scale forest monitoring, though further adaptation is needed to handle complex backgrounds effectively. Xiaotong Dong et al. [7] developed an optimized detection model based on YOLOv5s, introducing six different attention mechanisms that significantly improve feature extraction capabilities for diseased trees. The inclusion of randomized background images further enhances the model’s generalization ability. In addition to YOLO-based approaches, other deep learning architectures with attention mechanisms have also been explored. Dong Ren et al. [8] proposed a novel deep learning network called MASFNet for detecting PWD-infected trees. This model expands the receptive field via a multi-level attention mechanism to capture global contextual features from multiple dimensions. A spatial sampling fusion module is used to mitigate false positives caused by feature similarity.

In practical applications, PWD detection models often face deployment constraints on edge computing platforms or other resource-limited devices. To address these needs, some studies have focused on model lightweighting and improving small-object detection capabilities. Shikuan Wang et al. [9] introduced an improved YOLOv8-based rapid detection algorithm for PWD-infected trees. To enhance performance on small-scale trees, the model adds a dedicated small-object detection layer and incorporates attention modules into the backbone to enrich deep feature extraction [10]. Quanbo Yuan et al. proposed a lightweight PWD detection method based on a YOLO-enhanced Vision Transformer (ViT) [11]. This method utilizes a lightweight Multi-Scale Attention (MSA) module to construct an efficient ViT-based feature extraction network. The MBConv module is introduced to compensate for MSA’s limited capacity in capturing local details. Furthermore, a novel feature fusion network, CACSNet, replaces the PANet structure in YOLOv5 to prevent feature loss in small-object detection tasks, significantly improving performance on forest remote sensing images [12,13].

Beyond the YOLO framework, alternative deep learning architectures have been explored to diversify detection approaches for PWD. For example, Gensheng Hu et al. [14] proposed a method based on Deep Convolutional Neural Networks (DCNNs), combining MobileNet with Faster R-CNN to distinguish infected from healthy pine trees while minimizing background interference. Chen [15] developed a lightweight early-stage PWD detection model by integrating ViT and CNN, aiming to combine the strengths of both architectures for efficient and accurate early disease detection.

Although most existing studies rely on visible-light imagery and UAV platforms for detection, researchers have begun to investigate more diverse data sources and methods to enhance detection accuracy and early-stage identification. In PWD remote sensing tasks, the integration of deep learning with UAV-based multispectral imagery [16,17,18] has shown promise in improving early lesion detection. Recent studies have also utilized hyperspectral imagery [19,20,21] to capture fine-grained features, although computational complexity remains a challenge. Yuan [22] proposed using frequency-domain features to enhance detection robustness by reducing the influence of lighting and background variations, offering theoretical innovations in the field.

In summary, most existing studies have been conducted to improve the detection performance of PWD-infected trees; however, many issues remain unresolved. For instance, the generalization ability of the deep learning-based automatic detection models is weak, and the recognition precision in densely distributed or complex pine forests with overlapping crowns in remote sensing images is low. It is easy to confuse red broad-leaved trees with diseased and withered pine trees. The recognition methods are inevitably affected by occlusion between different objects, changes in illumination and shadow, and phenomena such as “same object, different color spectra” and “different objects, same spectrum”, leading to various misdetections and omissions, thereby affecting recognition accuracy. In our research, we proposed YOLOv8-MFD to reduce the false detection rate and utilized UAV remote sensing models for PWD detection. The main contributions of this study are as follows:

(1) Based on the UAV platform, we constructed a high-quality dataset of PWD that included early, middle, late, and death stages of disease characterization for both small and large targets.
(2) We propose a method that integrates the MobileViT feature extraction network into the backbone of the YOLOv8 network, enabling it to extract both local details of the target and capture extensive contextual information. This allows the model to adapt to complex environments and minimize background interference, better distinguishing between red broad-leaved trees and diseased and withered pine trees. The incorporation of the Focal Modulation module effectively mitigates the impact of uneven illumination by fusing distant context information. Additionally, the Dynamic Head’s dynamic adjustment capability ensures that the model can adapt flexibly to targets of varying scales, thereby significantly reducing errors resulting from differences in target shapes or sizes.
(3) In this study, a comprehensive evaluation was conducted on a homemade PWD dataset. The extent to which each module contributes to the model was verified through numerous ablation experiments, while the validity and superiority of our proposed model were confirmed through comparative experiments.

## 2. Materials and Methods

### 2.1. Study Area

**Figure 2:**
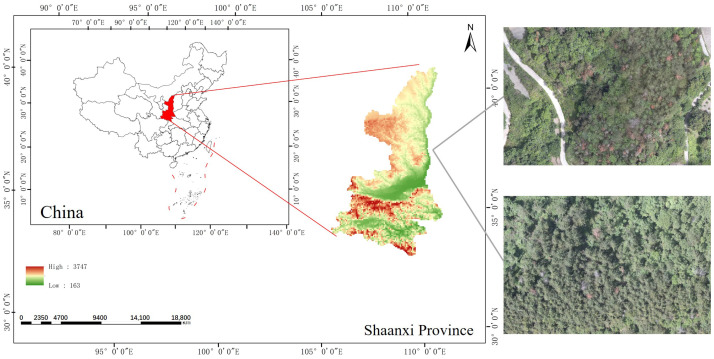
Location and snapshots of the research area.

The study area is located in Shaanxi Province, China, where Pine Wilt Disease (PWD)-infected trees serve as the primary research subjects. This region features a subtropical monsoon climate, with an average annual temperature of approximately 14 °C and an annual precipitation ranging from 800 to 1000 mm. The dominant tree species in the area are *Cunninghamia lanceolata* (*China fir*) and *Abies* spp. (firs), both belonging to the *Pinaceae* family. PWD is the most prevalent forest disease in this region. In this study, aerial imagery data were collected using unmanned aerial vehicle (UAV) technology, as depicted in Figure 2.

### 2.2. Data Collection and Annotation

This study used a UAV (Unmanned Aerial Vehicle) to conduct a cruise over the forested area in Shanxi Province, China, to collect high-resolution RGB images equipped with a Global Positioning System (GPS). These data are crucial for accurately identifying and assessing signs of PWD, aiding in the timely implementation of control measures to contain the outbreak. To acquire the necessary data for this research, we conducted multiple drone flights, covering an approximate total area of 1000 km². During the data collection process, a DJI UAV equipped with an onboard camera was used as the flight platform. The UAV operated at altitudes ranging from 80 to 300 m. Data were collected from July to September 2024, the optimal period when most PWD-infected pine trees exhibit typical reddish-brown symptoms [23], while the color of other broad-leaved trees remains unchanged. Considering the impact of light variation, altitude fluctuations, vegetation diversity, the complexity of areas with houses, rivers, and roads, and color differences at various stages of PWD, ensured the diversity and comprehensiveness of the data. We collected nearly 50 images and selected six typical ones to serve as the dataset for this experiment. Six orthoimages from different regions were generated with Pixel sizes of 37,256 × 45,079 pixels, 43,790 × 33,858 pixels, 46,742 × 38,909 pixels, 34,807 × 37,098 pixels, 41,884 × 40,646 pixels, and 43,939 × 36,080 pixels, respectively, and the geographical coordinate system of these image is WCG84. The details of these datasets are shown in Table 1.

**Figure 3:**
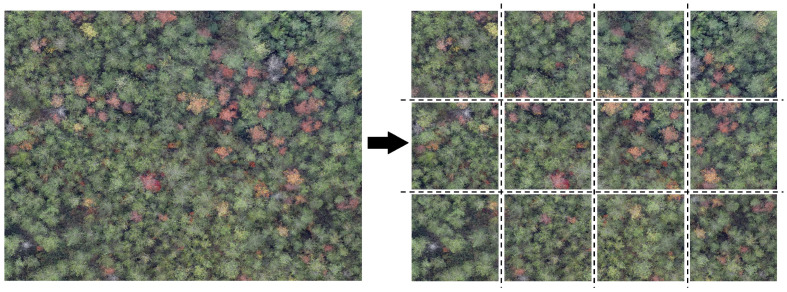
Diagram showing examples of image cropping.

This study utilized 5436 original UAV-acquired images of Pine Wilt Disease (PWD)-infected trees as the base dataset. Initially, we crop the original image into multiple sub-images, each measuring 640 × 640 pixels, as depicted in Figure 3.

**Figure 4:**
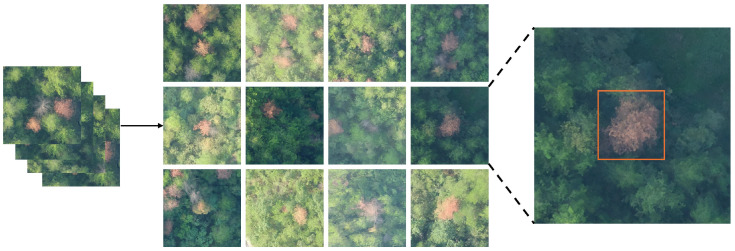
Diagram showing examples of data processing and dataset annotation.

To enhance the model’s generalization ability and mitigate overfitting under limited sample conditions, a comprehensive data augmentation strategy was employed, expanding the dataset to four times its original size, resulting in a total of 21,752 images. The specific augmentation techniques are as follows:

1. Geometric transformations: Horizontal and vertical flipping were applied to generate mirror images, thereby enriching the spatial diversity of the dataset;
2. Photometric adjustments: Linear brightness modifications were introduced to simulate varying illumination conditions, including brightness attenuation (factor 0.7) and enhancement (factor 1.2);

All augmentation procedures were implemented using the PIL and OpenCV libraries. The augmented dataset underwent dual quality control by a team of forestry experts: initially through visual inspection to remove distorted or low-quality samples, followed by precise annotation of PWD-infected trees using the LabelImg tool, as illustrated in Figure 4. The resulting labeled dataset includes 21,774 annotated instances of PWD-infected trees. The dataset was randomly partitioned into training and test sets at a ratio of 8:2. The training set comprises 16,552 images (corresponding to 4138 original images), while the test set consists of 5191 images (from 1298 original images), ensuring reliable and consistent model evaluation. This augmentation strategy substantially increases sample diversity and enhances model robustness, enabling better adaptability to variations in lighting conditions and complex background environments.

### 2.3. Improved YOLOv8 by MFD

#### 2.3.1. YOLOv8 as Basic Algorithm

**Figure 5:**
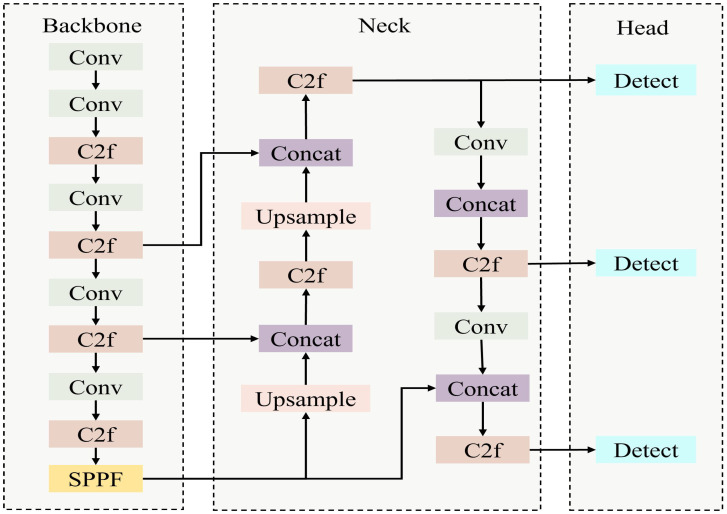
Diagram showing examples of YOLOv8 architecture.

YOLOv8 [24] is a YOLO-series object detection model released by Ultralytics in January 2023. The YOLOv8 architecture mainly consists of three parts: the backbone network, the feature fusion network (Neck), and the detection head (Head), and its structure is shown in Figure 5.

By incorporating the lightweight C2f module, YOLOv8 not only preserves the advantage of a compact model but also provides a richer flow of gradient information. Regarding the backbone network, YOLOv8 employs the optimized CSPDarknet53 architecture as a feature extractor and integrates the SPPF (Spatial Pyramid Pooling Fixed) module at the end of the backbone network to ensure the model’s compactness. In the design of the detection head, YOLOv8 introduces the concept of a Decoupled Head, which involves handling classification and regression tasks independently. YOLOv8 boasts efficient object detection capabilities and is extensively utilized in real-time detection and high-precision tasks. In this paper, YOLOv8 is used as the foundational model for detecting Pine Wilt Disease trees. Nevertheless, during research on utilizing YOLOv8 for the detection of Pine Wilt Disease trees, it was discovered that this method can be susceptible to interference from background noise, leading to misjudgments; it is not sufficiently accurate in detecting small targets or those that are similar to the background; and due to reliance on local features, it fails to fully capture global information in tasks involving detailed diseased tree detection, thereby impacting detection accuracy. Consequently, this paper proposes improvements to YOLOv8 to address these issues.

#### 2.3.2. MobileViT Backbone Module Principles

**Figure 6:**
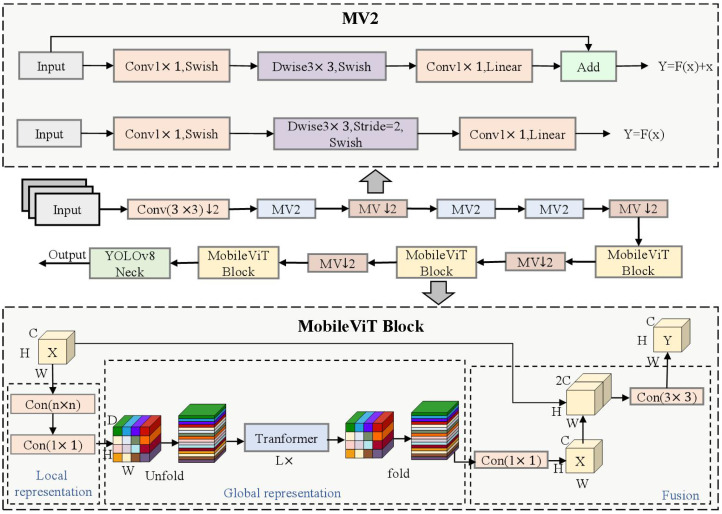
Diagram of MobileViT and the module network architecture.

Concerning the issue of YOLOv8’s subpar performance in capturing global features, which leads to reduced detection accuracy on remote sensing images of pine wood nematodes, this paper proposes the use of MobileViT to enhance the backbone network. MobileViT integrates the strengths of both CNN and Transformer architectures. It is capable of extracting local features as well as capturing global information, effectively diminishing background noise interference, and thus enhancing detection accuracy. The global feature extraction module, based on MobileViT [25], is situated within the Backbone section of the architecture and primarily consists of Convolutional (Conv), MobileViT v2 (MV2), and MobileViT Block components. The network structure diagrams for MobileViT and each module are depicted in Figure 6.

The MV2 module is the foundational module of MobileViT, derived from the inverted residual structure in MobileNetv2 [26]. It consists of depth-separable convolution, an activation function, and batch normalization. This module effectively preserves the non-linear expression ability of the feature map by initially expanding its dimension and then contracting it. Moreover, it substantially decreases the quantity of model parameters while achieving efficient feature extraction. The MobileViT module integrates convolution with the Transformer architecture, encompassing local feature modules, global feature modules, and fusion feature modules. These are employed to encode the local and global information within the input feature map efficiently. The Unfold and Fold operations are respectively utilized to transform the feature map into a vector format suitable for Transformer modeling and to revert the modeled vector back to the original feature map shape. After merging the local and global features with the original feature map, the fusion feature module reduces the dimensionality using an n×n convolution block, maintains the channel count, and forwards it to the subsequent module. This design not only maintains the local feature extraction capability of convolution but also achieves global feature representation.

#### 2.3.3. Focal Modulation SPPF Module Principles

**Figure 7:**
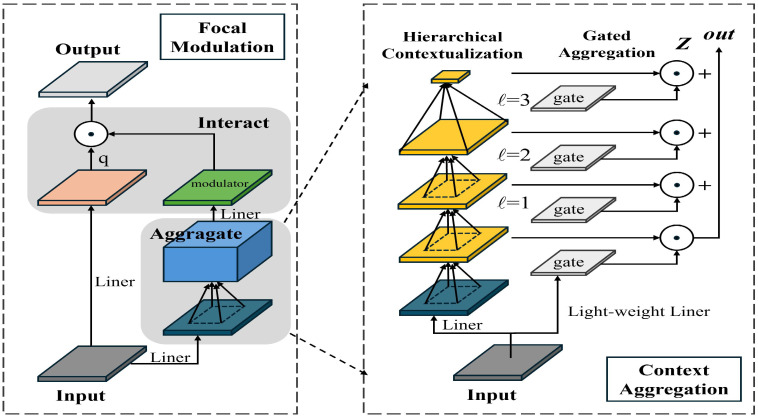
Diagram of Focal Modulation module architecture.

Addressing the issues of interference from background noise and inaccurate detection of targets that are similar to the background, this paper presents the Focal Modulation module to enhance the SPPF. The SPPF (Spatial Pyramid Pooling-Fast) module in YOLOv8 is primarily utilized to expand the model’s receptive field while keeping computational costs low. Nevertheless, the SPPF struggles to effectively capture global information and has a limited capacity to model long-range dependencies. This paper proposes the integration of the Focal Modulation module. By introducing the Focal Modulation Layer (FML), this module more efficiently models global information. The FML extracts initial features through local receptive fields and employs modulation operations to model global information. Compared to the traditional Transformer structure, Focal Modulation [27] achieves a lower computational cost while preserving the ability to capture global information. The structure of Focal Modulation is depicted in Figure 7.

After replacing the SPPF module with Focal Modulation in YOLOv8, the model’s global information modeling ability is enhanced, enabling the detector to better understand the relationships between targets. For instance, it can establish a connection between trees and their shadows cast by sunlight in the afternoon, reducing the misidentification of shadows.

#### 2.3.4. Dynamic Head Module Principles

To address the issue of YOLOv8’s lack of accuracy in detecting small targets within remote sensing images of pine wood nematodes, this paper proposes enhancements through the use of the Dynamic Head. This detection head dynamically optimizes the prediction structure based on the unique characteristics of the target, significantly enhancing the accuracy of target detection.

**Figure 8:**
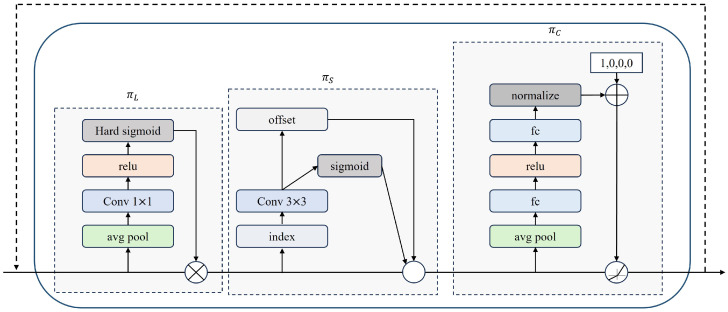
Diagram of Dynamic Head module architecture.

The module architecture of the Dynamic Head [28], henceforth referred to as DyHead, is depicted in Figure 8. DyHead represents a novel type of target detection head designed to simultaneously address three key issues: scale awareness, spatial awareness, and task awareness. It enhances feature representation by applying attention mechanisms across various dimensions (scale, spatial, and channel) of the feature tensor: scale-aware attention modifies features of different scales along the scale dimension, spatial-aware attention refines position features in the spatial dimension, and task-aware attention adjusts feature channels for various tasks (such as classification, regression, etc.) on the channel dimension. This strategic design effectively boosts target detection performance and can achieve a substantial Average Precision (AP) improvement across multiple tasks.

In Figure 8, $\pi_{L}$ represents scale-aware attention, $\pi_{s}$ represents spatial-aware attention, and $\pi_{c}$ represents task-aware attention. DyHead encompasses three primary attention modules. Among these, scale-aware attention is introduced first. This module applies attention to the scale dimension of features to discern the importance of different scales and to enhance the features pertinent to objects of varying sizes. By employing deformable convolution to sparsely sample features across different scales, the model can more effectively concentrate on features that are appropriate for the current object scale. The calculation formula of $\pi_{L}$ is illustrated in Equation (1):

(1) $$\pi_{L}\left(F\right)\cdot F=\sigma \left(f\left(\frac{1}{SC}\sum_{S,C}F\right)\right)\cdot F$$

Among them, f(·) represents a linear function approximated by a 1×1 convolutional layer.

Additionally, there is a spatial-aware attention module that is based on fused features. This module applies attention to the spatial dimension of the features (i.e., the height and width of the image), emphasizing spatial positions with high distinctiveness. Through self-learning of spatial offsets, the model can adaptively adjust the focus area to enhance the recognition ability of object positions. The calculation formula of $\pi_{s}$ is illustrated in Equation (2):

(2) $$\pi_{S}\left(F\right)\cdot F=\frac{1}{L}\sum_{l=1}^{L}\sum_{k=1}^{K}w_{l,k}\cdot F\left(l;p_{k}+\Delta p_{k};c\right)\cdot \Delta m_{k}$$

Among them, *K* represents the number of sparse sampling positions, while $p_{k}+\Delta p_{k}$ denotes the moving position of the self-learned spatial offset $\Delta p_{k}$, which is used to focus on the discriminative regions.

The task-aware attention module applies attention in the channel dimension of features, dynamically activating different feature channels based on the specific tasks (such as classification, regression, etc.). By controlling the activation of feature channels, the model can process feature representations more accurately for different tasks. The calculation formula of $\pi_{c}$ is illustrated in Equation (3):

(3) $$\pi_{C}\left(F\right)\cdot F=\max\left(\alpha^{1}\left(F\right)\cdot F_{c}+\beta^{1}\left(F\right),\alpha^{2}\left(F\right)\cdot F_{c}+\beta^{2}\left(F\right)\right)$$

Among them, $F_{c}$ represents the feature slice of the c-th channel, and ${\left[\alpha_{1},\alpha_{2},\cdot \beta_{1},\cdot \beta_{2}\right]}^{T}=\cdot \theta \left(\cdot \right)$ is a hyper-function for learning and controlling the activation threshold.

Since the aforementioned three attention mechanisms are applied sequentially, multiple $\pi_{L}$, $\pi_{S}$, $\pi_{C}$ modules are effectively stacked together through the following formula. The calculation formula of W(F) is illustrated in Equation (4):

(4) $$W\left(F\right)=\pi_{C}\left(\pi_{S}\left(\pi_{L}\left(F\right)\cdot F\right)\cdot F\right)\cdot F$$

Among them, $\pi_{L}$, $\pi_{S}$, and $\pi_{C}$ are three distinct attention functions applied to dimensions *L*, *S*, and *C*, respectively.

#### 2.3.5. Fusion of YOLOv8 and MFD PWD Detection Method

**Figure 9:**
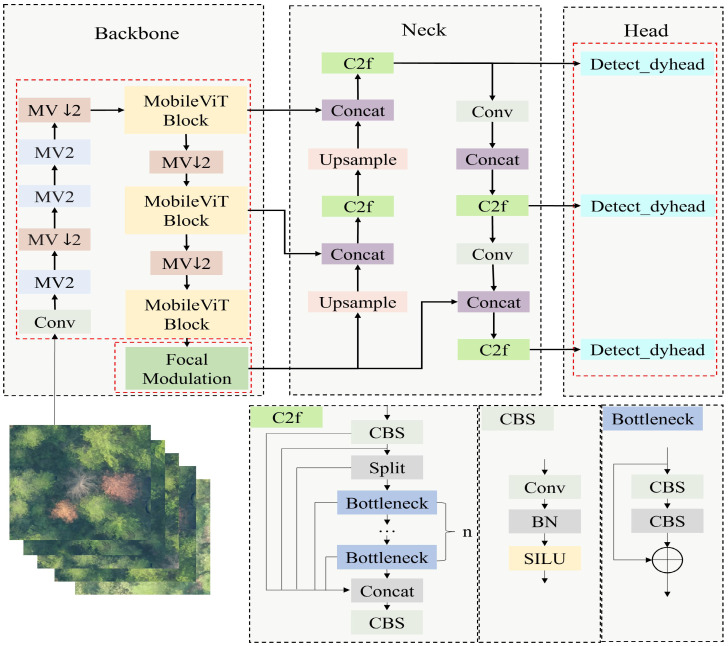
Diagram of YOLOv8-MFD model network architecture., where the section enclosed by the red dashed box represents the module area improved in this study.

In summary, this paper introduces an enhanced diseased-tree target detection model, YOLOv8-MFD, which is based on YOLOv8. The improved method optimizes the Backbone, SPPF, and Head, thereby enhancing the PWD detection performance and computational efficiency. The model structure is illustrated in Figure 9.

Firstly, the Backbone employs MobileViTv2. This module integrates the strengths of CNN and Transformer architectures, thereby improving the model’s feature extraction capabilities. With its lightweight global interaction mechanism, MobileViTv2 more effectively extracts both local and global information, enhancing the model’s ability to represent small targets and long-distance dependencies. At the feature fusion stage at the end of the Backbone, this study implements Focal Modulation in place of SPPF to enhance the model’s global information-capturing ability. Focal Modulation mitigates background noise interference while preserving the benefits of the local receptive field by introducing a modality interaction mechanism. It is particularly well suited for scenarios with complex backgrounds and significant scale variations, such as those found in remote sensing images. Finally, in the detection head section, this study incorporates DyHead to enhance the detector’s feature adaptability. DyHead allows features at various levels to adaptively select the optimal weight distribution via the Dynamic Convolution mechanism, thereby improving the accuracy of multi-scale detection. Additionally, this detection head is better equipped to handle targets of varying scales. This study presents an improved YOLOv8-MFD model. This model maintains computational efficiency while enhancing the global feature modeling ability and demonstrates superior detection accuracy and robustness in tasks involving small-target detection and complex-scene recognition.

### 2.4. Model Training and Evaluation

#### 2.4.1. Training Environment and Parameters

In the experiment, the Adam optimizer was selected to optimize the neural network. The initial learning rate was set to 0.001, and the learning rate decay factor was set to 0.01. During model training, the input image size was uniformly set to 640 × 640 pixels, the batch size was set to 16, and the number of epochs was set to 150. All experiments were conducted in the same experimental environment. The details of the experimental configuration are shown in Table 2.

#### 2.4.2. Evaluation Indicators

To meet the dual requirements of real-time performance and detection accuracy, this study adopts a comprehensive evaluation framework using the following metrics: mean Average Precision (mAP), *F*1-score, model size, GFLOPS, and FPS. The relevant calculation formulas are presented in Equations (5)–(8).

(5) $$\;Precision=\frac{TP}{TP+FP}$$

(6) $$\;Recall=\frac{TP}{TP+FN}$$

(7) $$\;F1=2\times \frac{Precision\times Recall}{Precision+Recall}$$

The F1-score comprehensively considers both the precision and recall of the model. For datasets with unbalanced class distributions, the F1-score can more objectively reflect the overall performance of the model.

(8) $$\;mAP=AP=\int_{0}^{1}P\left(R\right)\;dR$$

As a core metric in object detection tasks, mAP measures the average precision of the model across various IoU (Intersection over Union) thresholds, providing a comprehensive reflection of the model’s detection accuracy. A higher mAP value signifies that the model can more accurately locate targets and simultaneously reduce the likelihood of false positives and false negatives.

From the perspective of computational efficiency, model size determines the memory and storage required during inference. Smaller models are preferable for deployment on resource-constrained devices such as edge platforms. GFLOPS (Giga Floating Point Operations Per Second) measures the computational complexity of the model—higher GFLOPS implies greater inference cost. FPS (Frames Per Second) reflects the processing speed, indicating how many images the model can handle per unit time, and is a key indicator of real-time performance.

Therefore, in practical scenarios such as real-time forest monitoring or deployment on edge devices, it is essential to balance detection accuracy with computational efficiency. A well-optimized model should simultaneously ensure high detection precision and fast inference speed, fulfilling the dual demands of performance and deployment feasibility.

## 3. Results

### 3.1. Performance Comparison of Backbone Networks in YOLOv8

MobileViTv2 ingeniously integrates the local feature extraction capability of MobileNet with the global semantic modeling advantages of Vision Transformer, achieving a dynamic and efficient fusion of multi-scale contextual information. The deformable convolution module endows the model with the ability to adaptively adjust its receptive field, enabling effective capture of detailed features in dense small-object scenarios. Meanwhile, the sparse attention mechanism efficiently allocates computational resources, reducing computational complexity while maintaining long-range dependency modeling capability.

**Figure 10:**
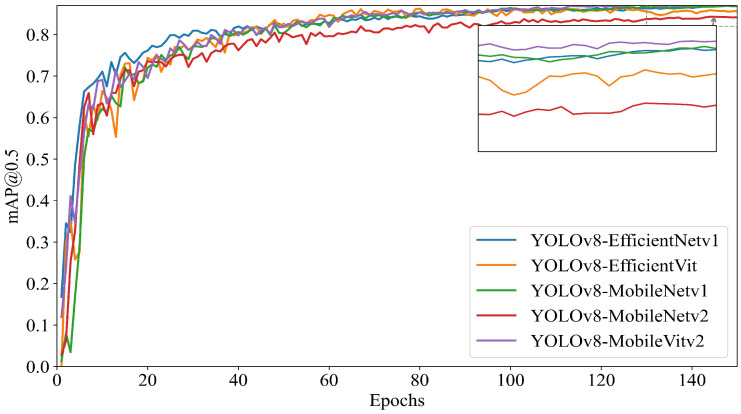
Comparison of mAP@0.5 across different backbone networks.

To verify the effectiveness of improving the YOLOv8 backbone network with MobileViTv2, this study conducts comparative experiments using EfficientNetv1, EfficientViT, MobileNetv1, MobileNetv2, and MobileViTv2 for modifications to the YOLOv8 backbone network. The experimental results of various performance metrics are shown in Table 3, and the mAP@0.5 variation curve with epochs is illustrated in Figure 10.

The experimental results indicate that after improving the YOLOv8 backbone network with MobileViTv2, the model significantly outperforms the baseline model and other backbone variants in key performance metrics, including precision, recall, F1-score, and mAP@0.5.

As shown in Table 3, the YOLOv8-MobileViTv2 backbone network improves precision by 1.2%, recall by 3.9%, F1-score by 2.7%, and mAP@0.5 by 3.1% compared to YOLOv8. In Figure 10, it is clearly observed that after 150 training epochs, MobileViTv2 achieves a higher mAP@0.5 than other backbone networks. Meanwhile, YOLOv8-MobileViTv2 demonstrates excellent performance in terms of model size, with an increase of only 0.62 MB compared to the baseline YOLOv8-base, which is smaller than other backbone variants, while still outperforming YOLOv8-base and other backbone variants in key metrics such as mAP@0.5.

These results demonstrate that the proposed backbone network improvement method maintains a compact structure while achieving stronger detection capabilities, significantly enhancing detection performance with only a slight increase in model size. Future work will focus on further improvements based on YOLOv8-MobileViTv2.

### 3.2. Enhanced Multi-Scale Detection in YOLOv8-MobileViT

The Focal Modulation module is introduced to enhance the model’s capability in capturing global information. Additionally, DyHead is incorporated to improve the detector’s feature adaptability, allowing it to better adapt to targets of different scales.

To analyze the contribution of different components in the modified model, we conducted ablation experiments on the dataset. The results of these experiments clearly demonstrate that each modification brings performance improvements to the model. The results are shown in Table 4.

Table 4 presents the results of a series of ablation experiments based on the YOLOv8 model, evaluating the effects of three different improvement strategies on diseased tree detection in remote sensing images.

After incorporating MobileViT, we observed a slight improvement in precision, while recall and mAP@0.5 significantly increased. This indicates that MobileViT enhances the model’s ability to distinguish and detect diseased trees, particularly in detecting Pine Wilt Disease, a highly infectious epidemic, where its positive impact on recall is especially prominent.

After integrating the Focal Modulation module, the model benefits from contextualization, gated aggregation, and element-wise affine transformations, improving the modeling of label interactions in vision tasks. Consequently, mAP@0.5 is further enhanced. This strategy significantly improves detection accuracy in complex backgrounds, making the Focal Modulation module particularly beneficial for remote sensing images, which often contain intricate backgrounds.

Replacing the original detection head with DyHead resulted in slight improvements in precision, recall, and mAP@0.5. This is because DyHead leverages scale-aware, spatial-aware, and task-aware attention mechanisms, effectively capturing multi-scale feature information. This advantage is particularly evident in processing complex remote sensing images of forests. These results demonstrate that enhancing YOLOv8 with various modules significantly improves the accuracy of diseased tree detection in forest remote sensing imagery.

**Figure 11:**
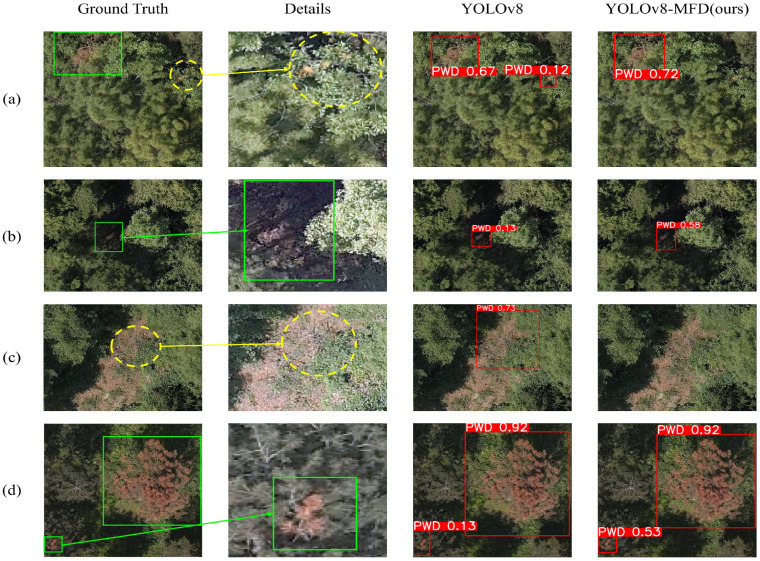
Diagram of YOLOv8 and YOLOv8-MFD comparison of inference results in different scenarios.Subfigure (**a**) shows a PWD-infected tree partially occluded by a discolored broadleaf tree. Subfigure (**b**) depicts a diseased tree located in a shadowed forest region. Subfigure (**c**) illustrates a diseased tree with a color similar to yellow exposed soil. Subfigure (**d**) presents two PWD-infected trees at different disease stages, showing varying discoloration areas. From left to right in each row: the original image with expert annotations (“Ground Truth”), zoomed-in details (“Details”), detection results of YOLOv8, and results of YOLOv8-MFD. green solid boxes indicate correctly detected diseased trees, while yellow dashed boxes represent false detections.

As shown in Figure 11, the detection performance of YOLOv8-MFD is compared with that of YOLOv8 under challenging conditions.

In the first row (a), a PWD-infected tree is partially occluded by a discolored broadleaf tree, which has a similar color to the diseased tree. YOLOv8 mistakenly identifies the small twigs of the broadleaf tree as diseased trees due to color similarity, leading to false detections. However, the proposed YOLOv8-MFD, utilizing the Focal Modulation module and DyHead detection head, enhances multi-scale feature extraction, enabling better discrimination between discolored non-PWD trees and diseased trees. Consequently, YOLOv8-MFD does not misidentify non-PWD regions, effectively reducing the false detection rate. Additionally, the introduction of the MobileViT module improves the confidence score for the PWD detection region from 0.67 to 0.72, further enhancing detection accuracy.

In the second row (b), the PWD-infected tree is located in a shadowed forest area where illumination variations and partial occlusion pose challenges for detection. The affected tree appears dark brown or black due to the shadow, making it prone to false negatives. While YOLOv8 manages to detect the tree, the detected edges are imprecise, and the confidence score remains low. In contrast, YOLOv8-MFD achieves more precise boundary detection and improves the confidence score from 0.13 to 0.58, effectively reducing the impact of shadows on detection accuracy.

For the third row (c), the image contains exposed yellowish soil that closely resembles the color of PWD-affected trees at the latest infection stage. Although no diseased trees are present in this region, the white withered shrubs within the yellow soil resemble late-stage PWD-infected trees, leading to a false detection with a confidence score of 0.73 using YOLOv8. However, YOLOv8-MFD correctly identifies the absence of PWD-affected trees, demonstrating its robustness in distinguishing visually similar regions and reducing false detections.

In the fourth row (d), there are two diseased trees at different infection stages. Early-stage infected trees exhibit minimal discoloration, making them difficult to detect. Detecting early-stage diseased trees as soon as possible is critical for controlling the spread of PWD and preserving forest ecology. Both YOLOv8 and YOLOv8-MFD successfully detect the two diseased trees, but YOLOv8-MFD provides more precise boundary detection. Notably, the confidence score for the early-stage infected tree improves from 0.13 to 0.53, significantly enhancing early-stage detection accuracy and reducing the likelihood of missed detections.

The experimental results indicate that YOLOv8-MFD maintains superior recognition accuracy under various complex environmental conditions, including occlusion, illumination variations, color similarity, and multi-scale targets at different infection stages.

### 3.3. Comparison of the Performance Metrics of Initial Models

To compare the recognition performance of the four YOLO series algorithms, this study conducts training and testing experiments using a self-built dataset. The table below presents a performance comparison among the YOLOv3, YOLOv5, YOLOv8, and YOLOv8-MFD models.

To comprehensively evaluate the effectiveness of the proposed model, we conducted comparative experiments with several mainstream object detection algorithms, including YOLOv3 [29], YOLOv5, YOLOv8, YOLOv9, YOLOv10, and RT-DETR. The evaluation focused on both detection accuracy and deployment feasibility, using metrics such as precision, recall, F1-score, mAP@0.5, model size, computational cost (GFLOPS), and inference speed (FPS). The results are presented in Table 5.

According to the results in Table 5, the proposed model achieves the highest F1-score (0.884) and mAP@0.5 (0.882), indicating a superior ability to balance precision and recall under complex remote sensing conditions. Compared to two high-performance baseline models, YOLOv10 and YOLOv9, our method improves the F1-score by 1.4% and 1.3%, respectively. Notably, the proposed model achieves a significantly higher recall (0.847) than YOLOv8 (0.799), suggesting enhanced sensitivity to infected trees under occlusion or low-contrast conditions.

In terms of model complexity and computational efficiency, although the proposed model exhibits a moderate increase in model size (10.2 MB) and GFLOPS (11.8), it remains within an acceptable range for UAV-based deployment. However, its inference speed (60.76 FPS) is lower than that of lighter models such as YOLOv5 (177.3 FPS) and YOLOv8 (164.38 FPS), primarily due to the introduction of additional modules aimed at improving robustness and context-awareness. This reflects a trade-off between accuracy and efficiency, which is justifiable given the task’s emphasis on reliable detection in noisy aerial environments.

Among the compared models, RT-DETR performs the weakest across all metrics; while YOLOv5 and YOLOv8 provide excellent efficiency, they offer slightly lower accuracy. YOLOv9 and YOLOv10 demonstrate better balance but are still outperformed by the proposed method.

These results validate the effectiveness of the proposed YOLOv8-MFD improvements in addressing challenges such as overlapping canopies, background interference, and multi-scale object detection—particularly for trees affected by Pine Wilt Disease.

## 4. Discussion

### 4.1. Model Innovations and Advantages

This study proposes YOLOv8-MFD, an improved object detection model based on YOLOv8. By integrating the MobileViT, Focal Modulation, and DyHead modules, the model significantly enhances the detection capability of pine trees during the discoloration stage caused by Pine Wilt Disease. The MobileViT module improves feature representation quality by balancing local and global modeling capabilities; the Focal Modulation module strengthens the model’s discriminative ability in complex backgrounds; and the DyHead module optimizes the model’s adaptability to multi-scale targets, especially small ones. Experimental results show that YOLOv8-MFD outperforms YOLOv8 in terms of mAP@0.5 and recall, with improvements of 4.0% and 4.8%, respectively. Additionally, the model demonstrates excellent accuracy and practicality in early-stage disease detection.

### 4.2. Limitations and Future Work

#### 4.2.1. Limitations

However, the model still exhibits certain limitations in identifying trees in the final (fourth) stage of Pine Wilt Disease, namely, the death stage. Trees in this stage have typically shed their needles, leaving only pale, dry branches with minimal distinguishing visual features. Additionally, their appearance closely resembles that of non-pathological dead trees, which increases the difficulty of accurate identification and leads to a higher risk of misclassification. Furthermore, samples from this stage are underrepresented in the training dataset, which may further constrain the model’s learning capacity.

**Figure 12:**
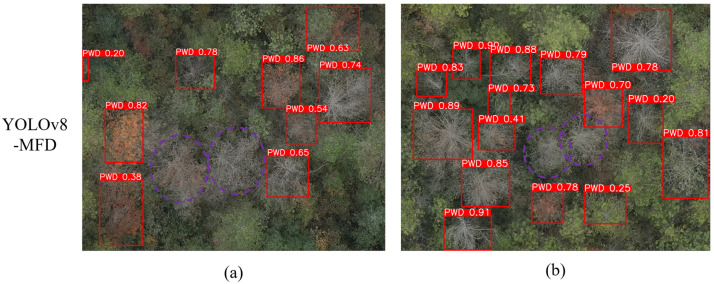
Examples of missed detections for dead-stage trees with white branches. Subfigures (**a**,**b**) show typical false negative cases, where clusters of closely adjacent trees in the death stage were not detected by the model. Dashed purple circles indicate the undetected regions.

In Figure 12, the regions highlighted with purple dashed circles illustrate false negative cases, where the model fails to detect clusters of closely adjacent dead-stage trees. This is likely caused by the dataset imbalance issue. To address this, additional dead-stage tree images will be collected and added to the dataset in future work to improve sample diversity. Figure 12 presents a typical missed detection example at this stage, highlighting the model’s current weakness in handling extreme categories. To further enhance generalization, future work will focus on augmenting the representation of death-stage samples to mitigate the class imbalance problem.

#### 4.2.2. Future Work

Future improvements can be made along two critical dimensions: data-level enhancement and algorithmic refinement.

From the data perspective, two main directions can be pursued: (1) Improved sampling techniques: Future studies should prioritize expanding the sample size of trees in the death stage to address class imbalance. In addition, more effective oversampling strategies—such as guided synthetic sampling based on class distribution characteristics—can be employed to avoid overfitting caused by naive duplication of minority class samples. (2) Data augmentation and generation: More effective data augmentation methods can be designed based on the phenotypic characteristics of diseased trees, such as rotation and noise injection. Moreover, targeted augmentations tailored to the unique properties of late-stage samples can be considered. To further enhance model robustness, generative approaches, such as Generative Adversarial Networks or conditional GANs, can be utilized to synthesize minority class instances.

From the algorithmic perspective, future efforts can focus on: (1) Incorporating spatial relational modeling: To better leverage environmental context, Graph Neural Networks could be employed to represent spatial relationships between the target and surrounding objects. Integrating context-aware mechanisms would enhance the model’s ability to interpret environmental cues—particularly valuable in the death stage, where visual features of infected trees often resemble those of naturally dead ones. Modeling spatial and semantic dependencies is expected to improve the recognition of weak-feature targets and reduce both false negatives and false positives. (2) Multimodal fusion: Establishing a multimodal detection framework by integrating multi-source remote sensing data would enable the model to capture subtle physiological and structural variations that are difficult to discern from standard RGB imagery. This fusion strategy can significantly enhance the model’s ability to identify trees in the death stage with higher precision.

### 4.3. Practical Applications and Generalization Potential

From a practical standpoint, the YOLOv8-MFD model is particularly suitable for the early detection and large-scale monitoring of Pine Wilt Disease. As the disease primarily spreads through vector insects such as *longicorn* [30], the early identification of infected trees in the “color-change” stage is critical for halting further transmission. By leveraging remote sensing images captured via unmanned aerial vehicles, the proposed model enables rapid localization of diseased trees, especially in complex terrains or areas inaccessible to manual inspection. This significantly enhances monitoring efficiency and emergency response capabilities. In real-world deployments, forest management personnel can utilize routine UAV patrols in conjunction with the proposed model for automated detection, allowing for timely removal of infected trees. This not only reduces the risk of disease spread but also lowers the time and labor costs of manual inspection, thereby improving the overall effectiveness of disease prevention and offering strong technical support for ecological protection.

Moreover, the model framework exhibits good transferability. With appropriate annotation and retraining, YOLOv8-MFD has the potential to be extended to the identification of other types of forest diseases—particularly those that exhibit distinct visual symptoms in remote sensing imagery, such as leaf spot or early-stage chlorosis.

## 5. Conclusions

This study proposes an improved remote sensing object detection model, YOLOv8-MFD, based on the YOLOv8 framework for efficient identification and monitoring of Pine Wilt Disease. The model introduces MobileViT as the backbone network, combining the efficient feature extraction capabilities of convolutional neural networks with the global modeling advantages of Vision Transformers, significantly enhancing the recognition performance of diseased areas. By integrating the Focal Modulation mechanism, the model’s discriminative power under complex backgrounds is strengthened, while the incorporation of the DyHead module in the detection head further optimizes multi-scale feature modeling, improving detection accuracy in remote sensing imagery. Empirical evaluations demonstrate that YOLOv8-MFD achieves a mAP@0.5 of 0.882 on the test dataset, representing a 4% improvement over the original YOLOv8 model, with only a 4.24 MB increase in model size. It demonstrates strong practicality in forest pest and disease detection scenarios.

Despite the model’s strong performance in detecting the early discoloration stage of Pine Wilt Disease, it still exhibits missed detections in later stages (particularly during the tree death phase), primarily due to the lack of distinct color and structural features, as well as limited training samples. Future work will focus on enriching death-stage samples and incorporating multispectral information to improve recognition in these more challenging cases.

In conclusion, YOLOv8-MFD offers an efficient and scalable solution for the automated monitoring of Pine Wilt Disease and holds significant practical value for large-scale forest disease surveys in complex and diverse terrain.

## Figures and Tables

**Table 1:** Information collected from six images.

Number	Date	Storage (GB)	Pixel Size
Image1	July 2024	1.61	37,256 × 45,079
Image2	July 2024	2.42	43,790 × 33,858
Image3	August 2024	2.97	46,742 × 38,909
Image4	August 2024	4.81	34,807 × 37,098
Image5	September 2024	6.34	41,884 × 40,646
Image6	September 2024	5.91	43,939 × 36,080

**Table 2:** Configuration of experimental environment.

Device Name	Configuration
Operating System	Windows 10
CPU	Intel Core i7-12700 (Intel Corporation, Santa Clara, CA, USA)
GPU	NVIDIA GeForce RTX 4060 Ti (NVIDIA Corporation, Santa Clara, CA, USA)
GPU Memory	16 GB
Programming Language	Python 3.11.5 (Python Software Foundation, Wilmington, DE, USA)
Framework	PyTorch 2.0.0 + cu118 (Meta AI, Menlo Park, CA, USA)
CUDA Version	12.6 (NVIDIA Corporation, Santa Clara, CA, USA)

**Table 3:** Performance comparison of different backbone networks.

Model	Precision	Recall	F1	mAP@0.5	Model Size (MB)
YOLOv8_Base	0.914	0.799	0.853	0.842	5.96
YOLOv8_EfficientNetv1	0.914	0.800	0.853	0.868	14.1
YOLOv8_EfficientViT	0.913	0.817	0.862	0.857	8.34
YOLOv8_MobileNetv1	0.911	0.808	0.856	0.869	11.8
YOLOv8_MobileNetv2	0.892	0.790	0.838	0.842	7.51
YOLOv8_MobileViTv2	**0.926**	**0.838**	**0.880**	**0.873**	6.58

Note: Bold values indicate the best results across each metric.

**Table 4:** Experimental results of different improvement ablation experiments on the optimal model.

Enhanced Modules			Performance Metrics				
MobileViT	Focal Modulation	DyHead	Precision	Recall	F1	mAP@0.5	Size/MB
×	×	×	0.914	0.799	0.853	0.842	5.96
✓	×	×	0.926	0.838	0.880	0.873	6.58
×	✓	×	0.889	0.792	0.838	0.847	6.17
×	×	✓	**0.931**	0.832	0.879	0.873	9.33
✓	✓	×	0.886	0.812	0.847	0.852	7.95
✓	×	✓	0.923	0.844	0.882	0.878	9.82
×	✓	✓	0.903	0.790	0.843	0.855	9.53
✓	✓	✓	0.925	**0.847**	**0.884**	**0.882**	10.2

Note: Bold values indicate the best results across each metric.

**Table 5:** Comparison of experimental results of different models.

Model	Precision	Recall	F1	mAP@0.5	Model Size (MB)	GFLOPS	FPS
YOLOv3	0.872	0.774	0.820	0.803	17.4	13	142.7
YOLOv5	0.902	0.806	0.851	0.837	**3.72**	**4.2**	**177.3**
YOLOv8	0.914	0.799	0.853	0.842	5.96	8.2	164.38
RT-DETR	0.655	0.621	0.637	0.622	66.2	108	50.42
YOLOv9	0.923	0.824	0.871	0.863	4.6	7.8	92.27
YOLOv10	0.924	0.806	0.860	0.857	5.8	8.4	144.36
Proposed	**0.925**	**0.847**	**0.884**	**0.882**	10.2	11.8	60.76

Note: Bold values indicate the best results across each metric.

## Data Availability

The data are not publicly available due to privacy.
